# The Relation of Having Experienced a Fall in the Past to Lower Cognitive Functioning in Old Age Is Mediated via Less Physical Activity Engagement as Cognitive Reserve Contributor

**DOI:** 10.3390/biology11121754

**Published:** 2022-12-01

**Authors:** Andreas Ihle, Élvio R. Gouveia, Bruna R. Gouveia, Adilson Marques, Priscila Marconcin, Marcelo de Maio Nascimento, Maximilian Haas, Jefferson Jurema, Maria A. Tinôco, Matthias Kliegel

**Affiliations:** 1 Department of Psychology, University of Geneva, 1205 Geneva, Switzerland; 2 Center for the Interdisciplinary Study of Gerontology and Vulnerability, University of Geneva, 1205 Geneva, Switzerland; 3 Swiss National Centre of Competence in Research LIVES—Overcoming Vulnerability: Life Course Perspectives, 1015 Lausanne, Switzerland; 4 Department of Physical Education and Sport, University of Madeira, 9020-105 Funchal, Portugal; 5 Laboratory of Robotics and Engineering Systems (LARSYS), Interactive Technologies Institute, 9020-105 Funchal, Portugal; 6 Regional Directorate of Health, Secretary of Health of the Autonomous Region of Madeira, 9004-515 Funchal, Portugal; 7 Saint Joseph of Cluny Higher School of Nursing, 9050-535 Funchal, Portugal; 8 Centre for the Study of Human Performance (CIPER), Faculty of Human Kinetics, University of Lisbon, 1495-751 Lisbon, Portugal; 9 Instituto de Saúde Ambiental (ISAMB), Faculty of Medicine, University of Lisbon, 1649-020 Lisbon, Portugal; 10 Faculty of Human Kinetics, University of Lisbon, 1495-751 Lisbon, Portugal; 11 KinesioLab, Research Unit in Human Movement Analysis, Piaget Institute, 2805-059 Almada, Portugal; 12 Department of Physical Education, Federal University of Vale do São Francisco, 56304-917 Petrolina, Brazil; 13 Higher School of Health Sciences, Amazonas State University, 69065-001 Manaus, Brazil; 14 Coordination of Physical Education and Sport, Federal Institute of Science and Technology Education of Amazonas, 69020-120 Manaus, Brazil

**Keywords:** physical activity, exercise, falls, mental health, cognitive performance, cognitive abilities, cognitive reserve, old age, life events

## Abstract

**Simple Summary:**

Experiencing a fall in old age represents a critical life event affecting physical and cognitive health and the ability to engage in physical activities and exercise. This is crucial since physical activity engagement contributes to the accumulation of the so-called cognitive reserve relevant for maintaining cognitive health at old age. The goal of our study was to investigate whether the relationship between having experienced a fall and lower cognitive functioning can be explained by hampered physical activity engagement. Confirming this idea, our findings demonstrated that experiencing a fall at an older age hinders sufficient physical activity engagement and thereby impedes cognitive reserve accumulation, resulting in lower cognitive functioning outcomes. Consequently, our study suggests that at old age, the prevention of falls and related accidents is not only crucial to avoid injuries and preserve physical health, but it is also essential for maintaining one’s ability to engage in physical activities and exercises and, consequently, for preserving cognitive health in later life.

**Abstract:**

Physical activity and exercise contribute to the accumulation of cognitive reserve, which is instrumental for preserving cognitive health in old age. In a large sample of 701 older adults (mean age = 70.36 years), we investigated whether the relationship between having experienced a fall in the past and lower performance in cognitive functioning was mediated via less physical activity engagement as a cognitive reserve contributor. General cognition was assessed using the mini-mental state examination (MMSE), long-term memory using a word-pair delayed recall test and working memory using a backward digit-span test. In face-to-face interviews, individuals reported information on falls during the past 12 months and their habitual physical activity engagement. Our analyses demonstrated that the relationship between having experienced a fall in the past and lower performance in the cognitive functioning measures was partly mediated (by 16.3% for general cognition, 30.6% for long-term memory, and 33.1% for working memory, respectively) via less physical activity engagement. In conclusion, we suggest as a core bio-psychological mechanism that experiencing a fall at an older age is a critical life event that hinders sufficient physical activity engagement and thereby impedes cognitive reserve build-up, resulting in lower cognitive functioning outcomes.

## 1. Introduction

Physical activity and exercise are important for the maintenance of health at old age because they are related to a reduced risk of developing chronic diseases, including heart diseases, cerebrovascular diseases, and metabolic syndromes, such as diabetes and obesity [1,2,3,4,5]. With the intention of combating sedentary lifestyles in the older population, the World Health Organization [6] suggested guidelines for the promotion and maintenance of health. Recommendations are to do at least 150 min of moderate-intensity aerobic activity per week, 75 min of vigorous-intensity aerobic activity per week, or seek an equivalent combination of both. Moreover, to prevent falls, it is recommended to additionally carry out activities for muscle strengthening and balance training three or more days per week. The mechanism that underlies this positive relationship between exercise, physical activity, and aging involves skeletal muscle, the immune and endocrine systems, gastrointestinal activity, as well as cognitive functioning [3,7]. Moreover, physical activity and exercise have protective effects on neurocognitive health, with different potential mechanisms involved [8]. For instance, possible molecular and cellular mechanisms include physical activity evoked increases in the availability of growth factors, such as the vascular endothelial growth factor, insulin-like growth factor-1, and brain-derived neurotrophic factor, that are related to enhanced neurogenesis, synaptogenesis, and brain plasticity [9,10,11,12]. Physical activity is further related to volume increases in the prefrontal cortex, hippocampus, and caudate nucleus, as well as augmented white matter integrity and improved functional connectivity [13,14,15,16,17,18,19,20,21]. Thereby, physical activities have a positive effect on preserving cognitive health, resulting in increased cognitive performance at old age [22,23,24,25,26,27,28].

A complementary mechanism underlying the beneficial relationships between physical activity and cognitive outcomes is that physical activity engagement contributes to the accumulation of cognitive reserve that is instrumental for preserving cognitive functioning at old age. In general, the cognitive reserve concept [29] postulates that certain activities over a lifespan, such as physical activity and exercise, build up a buffering potential that will later on in life help to compensate for neurological loss and pathological decline, such as dementia [30,31]. In non-clinical populations with healthy cognitive development, these enhancing mechanisms support the adaptation of brain activity and cognitive processes in situations of increased cognitive demands and, consequently, help to improve cognitive performance [30,31,32]. Empirical evidence corroborating these postulations of the cognitive reserve concept demonstrated that physical activity engagement, such as walking, gardening, sports, and exercises, contribute to the accumulation of cognitive reserve and are related to better cognitive functioning at old age as well as lower risk and later onset of pathological decline, such as dementia [33,34,35,36,37,38,39,40,41,42,43,44].

Importantly, with regard to physical activity and exercise, falls occur relatively often at an older age. Studies estimating fall prevalence reported that in the duration of a year approximately 25–45% of older individuals experience a fall [45,46,47,48,49,50,51]. This relatively high prevalence is alarming since falls often cause injuries, such as fractures and wrenches [52], which hinder sufficient physical activity engagement during the rehabilitation phase [53,54]. Moreover, from a long-term perspective, due to the fear of falling again, older people also reduce their physical activity engagement [55,56,57,58,59,60,61]. These reductions in physical activity engagement will then go along with a hampered accumulation of cognitive reserve [30,33,34,35,36,37,38,39,40,41,42,43,44]. Notably, the observation of a decline in cognitive functioning over several years following fall events [62] may be explained by the aforementioned detrimental influences of falls on subsequently hampered cognitive reserve build-up.

Thus, compiling the interplay of the abovementioned relationships into one overarching framework, we postulate a bio-psychological mediation mechanism in which experiencing a fall at old age will hinder sufficient physical activity engagement and thereby impede cognitive reserve build-up, which in turn will result in reduced preservation of cognitive functioning and lower performance in cognitive outcomes. However, to the best of our knowledge, this mediation mechanism has so far not been thoroughly verified in a large-scale study targeting the older population. Therefore, our study aimed to investigate in a large sample of older adults whether the relation of having experienced a fall in the past to lower performance in cognitive functioning (general cognition, long-term memory, and working memory) was mediated via less physical activity engagement as a cognitive reserve contributor. Based on the rationale detailed above, we hypothesized relations of having experienced a fall in the past to lower performance in cognitive functioning and to less physical activity engagement, a relation of less physical activity engagement to lower performance in cognitive functioning, and a mediation of the relationship of having experienced a fall in the past to lower performance in cognitive functioning via less physical activity engagement.

## 2. Materials and Methods

### 2.1. Sample and Study Design

The present cross-sectional study consisted of 701 older adults from Fonte Boa, Apuí, and Manaus in Brazil’s Amazonas region. All individuals participated in the project “Health, Lifestyle, and Functional Fitness in the Older People from Amazonas, Brazil” (SEVAAI), led by the Amazonas State University, Brazil, and the University of Madeira, Portugal. The SEVAAI project aimed to better understand the association between lifestyle and health in older adults from the Amazonas region of Brazil [63]. All individuals were community-dwelling volunteers recruited through advertisements distributed through churches, senior centers, local radio, and newspapers. The inclusion criteria for recruitment in this study were (1) residence in one of the three selected geographic regions of Brazil and (2) age 60 years or older. The SEVAAI study initially recruited 756 individuals meeting these two inclusion criteria. Of these, 55 individuals could not be further included because of comorbidities that would compromise the execution of the protocols, or individual drop-out before the assessments. Thus, 701 individuals were finally included in the study. Of these 701 participants, 433 were women, and 268 were men. Participants’ mean age was 70.36 years (*SD* = 6.87). The data were collected between July and December 2016. All individuals provided their informed consent before participating. The study adhered to the Declaration of Helsinki and had been approved by the local ethics committee before the start of the data collection (ethics committee name: The Research Ethics Committee—Human Beings; approval code: CAAE: 56519616.6.0000.5016, number: 1.599.258, Brazil Platform; approval date: 20 June 2016).

### 2.2. Instruments

#### 2.2.1. Cognitive Functioning Measures

General cognition. We administered the Portuguese version [64] of the mini-mental state examination (MMSE) [65] to assess general cognition. The MMSE covers a variety of basic abilities, including spatio-temporal orientation, memory (free recall of three words), arithmetic (counting backwards), and language (naming objects, understanding and following simple commands, etc.).

Long-term memory. We used the Portuguese version [66] of the word-pair delayed subtest of the Wechsler Memory Scale-Revised Edition (WMS-R) [67] to assess long-term memory. Individuals were asked to memorize eight pairs of words that were read aloud by the experimenter. During an interval of approximately 15 min, individuals filled out a socio-demographic questionnaire (including information about sex, age, and residence) and performed the working memory task. After this interval, the long-term memory recall test followed. The experimenter read aloud the first word of every word pair (in a different order than initially presented), and the individual had to recall the second word of each word pair (for validation and reliability evaluations of this test, see e.g., [66,67,68,69]).

Working memory. We administered the Portuguese version [70] of the backward digit-span subtest of the Wechsler Adult Intelligence Scale-Revised Edition (WAIS-R) [71] to assess working memory. Individuals listened to 12 progressively longer sequences of single-digit numbers that were read aloud by the experimenter. Their task was to immediately recall each sequence by repeating all respective digits in the reverse order in which they had been initially presented (for validation and reliability evaluations of this test, see e.g., [68,69,70,71]).

#### 2.2.2. Having Experienced a Fall in the Past

We asked individuals in face-to-face interviews whether they had experienced a fall during the past 12 months (yes/no).

#### 2.2.3. Physical Activity Engagement

We questioned individuals in face-to-face interviews regarding their habitual physical activity engagement as a contributor to cognitive reserve [30,33,34,35,36,37,38,39,40,41,42,43,44]. Specifically, they were asked “How would you classify your habitual physical activity engagement?”. Individuals made this classification based on a five-point Likert-type rating scale ranging from 1 (not active or sedentary) to 5 (very active). For similar items, see e.g., [72,73,74,75].

### 2.3. Statistical Analyses

**Figure 1:**
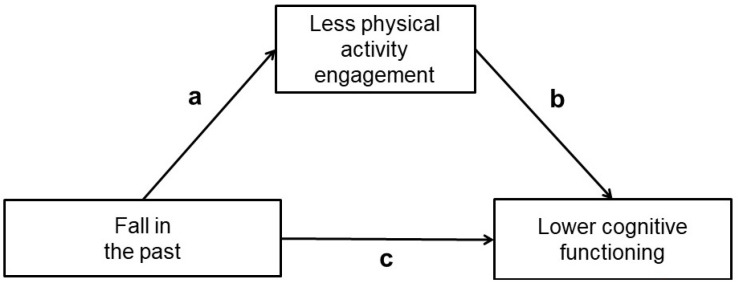
Illustration of the general analytical design of the mediation models applied to investigate whether the relation of having experienced a fall in the past to lower performance in the cognitive functioning measures was mediated via less physical activity engagement as a cognitive reserve contributor.

First, we evaluated bivariate relationships in terms of Pearson’s correlation coefficients *r*, except for the variable of having experienced a fall in the past, for which we calculated point-biserial correlation coefficients *r_pb_*. Regarding our core study objective, we applied mediation analyses [76,77,78] to examine whether the relationship of having experienced a fall in the past to lower performance in the cognitive functioning measures was mediated via less physical activity engagement as a cognitive reserve contributor (see Figure 1 for an illustration of the analytical design). Importantly, for investigating these mediational mechanisms, the applied analytical approach allowed to simultaneously estimate the residual direct (i.e., non-mediated) relationship between having experienced a fall in the past and lower cognitive functioning (i.e., the coefficient of the path c) and the indirect (i.e., mediated) relation via less physical activity engagement (i.e., the product of the coefficients for the paths a and b), while also computing their significance [76,77,78]. The data presented in this study are available online as Supplementary Materials (Table S1).

## 3. Results

### 3.1. Descriptive Statistics

The mean scores of the cognitive functioning measures were 24.41 (*SD* = 4.23) for general cognition, 4.50 (*SD* = 2.18) for long-term memory, and 3.52 (*SD* = 2.65) for working memory. Regarding falls, 32.4% of the sample reported that they had experienced a fall during the past 12 months. The mean score in physical activity engagement was 3.58 (*SD* = 1.24).

Regarding relationships between the investigated variables, having experienced a fall in the past was related to lower performance in the cognitive functioning measures and to less physical activity engagement. Moreover, less physical activity engagement was related to lower performance in the cognitive functioning measures (see Table 1 for an overview).

### 3.2. Mediation Analyses

As expected, the relation between having experienced a fall in the past and lower performance in the cognitive functioning measures was partly mediated (by 16.3% for general cognition, 30.6% for long-term memory, and 33.1% for working memory, respectively) via less physical activity engagement as a contributor to cognitive reserve (see Table 2 for an overview).

## 4. Discussion

In the present study, we investigated whether the relationship between having experienced a fall in the past and lower performance in cognitive functioning (general cognition, long-term memory, and working memory) was mediated via less physical activity engagement as a cognitive reserve contributor. The prevalence of falls during the past 12 months observed in our sample (approximately 30%) is comparable to other studies on falls at old age [46,48,49,50,51].

The bivariate relationships between the examined variables are in line with prior empirical research. Specifically, our observations that having experienced a fall in the past was related to lower performance in the cognitive functioning measures are consistent with previous evidence of a decline in cognitive functioning over several years following fall events [62]. Second, the relation of having experienced a fall in the past to less physical activity engagement observed in our study further corroborates previous research showing that falls lead to reductions in physical activities as a consequence of falling injuries and the fear of falling again [52,53,54,55,56,57,58,59,60,61]. Third, the relation of less physical activity engagement to lower performance in the cognitive functioning measures (respectively, greater physical activity engagement being related to better cognitive performances) found in our study is in line with research suggesting that, in general, physical activities have a positive effect on preserving cognitive health, resulting in increased cognitive performance in old age [22,23,24,25,26,27,28]. More specifically, these findings are consistent with the cognitive reserve concept [29,31] and empirical research documenting that physical activity and exercise contribute to the accumulation of cognitive reserve and are related to better cognitive functioning at old age [30,33,34,35,36,37,38,39,40,41,42,43,44].

Most importantly, concerning the novel contribution of arranging all these relationships into one overarching mediation framework, our analyses demonstrated that the relation of having experienced a fall in the past to lower performance in the cognitive functioning measures was mediated via less physical activity engagement. Accordingly, we emphasize a bio-psychological mediation mechanism in which experiencing a fall at old age will hinder sufficient physical activity engagement and impede cognitive reserve build-up, resulting in reduced preservation of cognitive functioning and, consequently, lower performance in cognitive outcomes.

Thereby, with regard to conceptual implications, our study suggests that experiencing a fall represents a critical life event that, by hampering sufficient physical activity engagement, negatively influences the individual’s pathway of cognitive reserve accumulation and, therefore, should be considered in cognitive reserve research. Moreover, our findings also have important practical implications. At old age, the prevention of falls and related accidents is not only crucial in avoiding injuries and preserve physical health, but it is also essential for maintaining one’s ability to engage in physical activities and exercises since they will help to preserve cognitive health in later life.

Regarding the limitations of our study, we acknowledge that its correlative cross-sectional design does not allow drawing conclusions regarding causality and changes over time. Importantly, regarding the temporal order of variables in our data, the investigated fall events refer to a time frame during the past 12 months prior to cognitive assessments. Likewise, since we were interested in habitual physical activity engagement (not activity currently at present), it referred to a period of sometime before the cognitive assessments, thus in most cases likely between fall events and cognitive assessments. Hence, the condition of temporal order is fulfilled. Nevertheless, the present study may stimulate further investigations in future longitudinal research to better understand causality and the direction of effects. Moreover, a part of the data was based on retrospective evaluations and self-reports. Retrospective reports of fall events have been confirmed as reliable and valid assessments of fall experiences in the past [45,46,47,48,49,50,51]. Likewise, as documented in a large body of empirical evidence, self-reports on physical activity engagement, such as walking, gardening, sports, and exercises, are reliable and valid contributors to cognitive reserve [33,34,35,36,37,38,39,40,41,42,43,44]. Furthermore, we agree that the single physical activity item used may not capture all aspects of physical activity. However, it is important to note that, in vulnerable older adults, as in the present study, long questionnaires are unfortunately hard to apply. Based on our experiences in the field and during study piloting, we saw that one simple question in this specific population is more straightforward for them to understand and answer, and does provide more reliable and valid results. This is one of the reasons why simple single items are used in large-scale surveys of older adults and other populations. Those items show good reliability and validity for capturing inter-individual differences in physical activity [72,73,74,75]. Likewise, in our correlative study, the single physical activity item used was able to capture a sufficient amount of inter-individual differences, as reflected, e.g., in the medium sized, significant associations between physical activity and the other variables.

Regarding the strengths of our study, to minimize bias in the assessment of fall events and physical activity engagement as a contributor to cognitive reserve, we conducted face-to-face interviews. Further important strengths include the detailed assessment of cognitive performance in a large sample of older adults.

## 5. Conclusions

We suggest as a core bio-psychological mechanism that experiencing a fall at an older age hinders sufficient physical activity engagement and thereby impedes cognitive reserve build-up, which in turn results in lower cognitive functioning outcomes. Hence, cognitive reserve research should consequently consider the occurrence of a fall as a critical life event because it adversely affects the individual’s pathway of cognitive reserve accumulation and thereby has negative consequences for preserving cognitive health at old age.

## Figures and Tables

**Table 1:** Relationships between analyzed variables.

	Fall	Physical Activity
General cognition	−0.14 ***	0.27 ***
Long-term memory	−0.10 **	0.35 ***
Working memory	−0.07 ’	0.27 ***
Physical activity	−0.09 *	---

*** *p* < 0.001; ** *p* < 0.01; * *p* < 0.05; ’ *p* < 0.10; significance at one-tailed level.

**Table 2:** Results of mediation analyses.

	Indirect Relation	Residual Direct Relation
General cognition	−0.02 * (16.3%)	−0.12 **
Long-term memory	−0.03 * (30.6%)	−0.07 ’
Working memory	−0.02 * (33.1%)	−0.05 ns

Results of mediation analyses to investigate whether the relation of having experienced a fall in the past to lower performance in the cognitive functioning measures was mediated via less physical activity engagement as a cognitive reserve contributor. Left panel: Values represent indirect (mediated) relation sizes β. In parentheses, the portion of the relation of having experienced a fall in the past to lower cognitive functioning that was exerted indirectly via less physical activity engagement is given. Right panel: Values represent residual direct (non-mediated) relation sizes β. ** *p* < 0.01; * *p* < 0.05; ’ *p* < 0.10; significance at the one-tailed level; ns = non-significant; *p* > 0.10.

## Data Availability

The data presented in this study are available online as Supplementary Materials (Table S1).

## Supplementary Materials

The following supporting information can be downloaded at: https://www.mdpi.com/article/10.3390/biology11121754/s1, Table S1: Dataset of analyzed variables.
